# Dataset of adulteration with water in coconut milk using FTIR spectroscopy

**DOI:** 10.1016/j.dib.2021.107058

**Published:** 2021-04-20

**Authors:** Agustami Sitorus, Muhamad Muslih, Irwin Syahri Cebro, Ramayanty Bulan

**Affiliations:** a Research Center for Appropriate Technology, Indonesian Institute of Sciences (LIPI), Subang, Indonesia; b Department of Information System, Nusa Putra University, Sukabumi, Indonesia; c Department of Mechanical Engineering, Lhokseumawe State Polytechnic, Lhokseumawe, Indonesia; d Department of Agricultural Engineering, Syiah Kuala University, Banda Aceh, Indonesia

**Keywords:** Adultration, Appropiate technology, Fourier transform infrared, Rapid detection

## Abstract

This paper presents the spectroscopic dataset, pre-processing, calibration, and predicted model database of Fourier transform infrared (FTIR) spectroscopy used to detect adulterated coconut milk with water. Absorbance spectral data were acquired and recorded in wavelength range from 2500 to 4000 nm for a total of 43 coconut milk samples. Coconut milk ware prepared in three forms of adulteration. Coconut milk comes from traditional markets and instant coconut milk in Indonesia. Spectra data may also be pre-processed to increase prediction accuracy, robustness performance using normalize, multiplicative scatter correction (MSC), standard normal variate (SNV), 1st derivative, 2nd derivative, and combination of 1st derivative and MSC. Calibration models and cross-validation to forecast those adulteration parameters use two regression algorithms, i.e., principal component regression (PCR) and partial least square regression (PLSR). By looking at its statistical metrics, prediction efficiency can be measured and justified (correlation coefficient (r), correlation of determination (R^2^), and root mean square error (RMSE)). Obtained FTIR datasets and models can be used as a non-invasive method to predict and determine adulteration on coconut milk.

## Specifications Table

**Table:** 

Subject	Agricultural and Biological Sciences
Specific subject area	FTIR Spectroscopy, non-invasive and fast adultration determination methodology
Type of data	Table Graph
How data were acquired	Infrared spectral data of coconut milk ware collected using a compact FTIR instrument (Bruker alpha II) in the wavenumber range from 3997 to 2500 cm^−1^ with 2.06 cm^−1^ resolution windows. The light source of the halogen lamp irradiated coconut milk samples through a quartz glass about 1 cm in diameter. Liquid sample coconut milk was placed manually in the crystal on top of the FTIR instrument. After each measurement, the crystal is cleaned by rinsing and wiping with alcohol 99%. The correction of background spectra was performed manually once every 5 sample acquisitions. In the presence of energies from 3997 to 2500 cm^−1^, raw spectral data was acquired and recorded as absorption spectrum and then converted to wavelength (2500–4000 nm) for a total of 43 samples from 2 types of coconut milk source. As an average of 43 consecutive spectra data acquisition, each spectral information consisted of 729 wavelength variables. Pre-processing datasets of spectra were obtained by transforming data of raw spectra using specified algorithms: normalize, multiplicative scatter correction (MSC), standard normal variate (SNV), 1st derivative, 2nd derivative, and combination of 1st derivative and MSC. This spectral dataset was used to construct prediction models to determine adulteration in coconut milk.
Data format	Raw Analyzed Presented as .xls extension file format
Parameters for data collection	Spectra coconut milk sample datasets were used to estimate water-added adulteration parameters. Coconut milk samples were collected from traditional markets whilst instant coconut milk products traded at the modern market in Indonesia.
Description of data collection	Infrared spectroscopic data as absorbance spectrum is used to forecast coconut milk adulteration. These models were built using the most usual technique: principal component regression (PCR) and partial least square regression (PLSR) followed with cross-validation to avoid overfitting models. Furthermore, forecasting models may also be built utilizing other regression methods such as non-linear regression methods and stepwise and backward linear regression. Spectral results, collected from normal laboratory measurements, were regressed with actually added water to coconut milk as adulteration. The predicted value of add water to coconut milk was then compared with the actual measured add water to coconut milk to evaluate model performances.
Data source location	Data from FTIR spectra and coconut milk for adulteration were collected at the Research Center for Appropriate Technology, Indonesian Institute of Sciences (LIPI), Subang, Indonesia
Data accessibility	The datasets are provided as extension formats for MS Excel (.xlsx) and are available in this report. Dataset was also stored in Mendeley's repository info: https://data.mendeley.com/datasets/byww6nrp2z/1 or http://dx.doi.org/10.17632/byww6nrp2z.2

## Value of the Data

• FTIR spectral dataset can be used to predict adulteration in coconut milk. It provides a non-invasive method to evaluation and quality inspection monitoring.
• FTIR spectra datasets can benefit for food inspectors to inspect every quality of coconut milk that is traded in the market.
• Using numerous spectra enhancement and regression algorithms, the FTIR spectra data and model database may be remodeled to improve prediction efficiencies such as support vector regression and artificial neural networks.
• The model database from spectral FTIR data can protect coconut milk consumers from the product's adulteration efforts.

## 1 Data Description

**Fig. 1:**
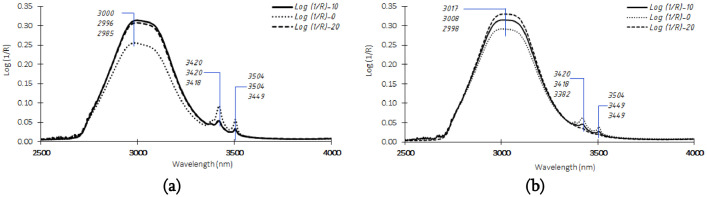
Typical FTIR of coconut milk absorbance spectrum come from (a) traditional markets and (b) instant coconut milk from modern market.

FTIR spectral data collection of coconut milk adulteration samples was acquired and reported as wavelength absorbance range from 2500 to 4000 nm (Fig. 1). The samples consisted of coconut milk from traditional markets and instant coconut milk from modern markets in Indonesia. Fig. 1a describes the spectral data of coconut milk from the traditional market before pre-processing. Three absorbance peaks occur in this type of coconut milk, namely in the wavelength range of 2985–3000 nm, 3418–3420 nm, and 3449–3504 nm. Furthermore, coconut milk absorbance peaks from modern markets occurred in the wavelength ranges of 2998–3017 nm, 3382–3420 nm, and 3449–3504 nm (Fig. 1a). Typically, infrared spectra data can be represented as a wavenumber (cm^−1^) or wavelength (nm) of the electromagnetic radiation. Spectral data contains chemical properties and information that can be known through chemometrics approaches. However, spectral data can also have irrelevant information, which is known as noise. One of the causes of this is light scattering. The existence of this noise can interfere with the accuracy of the predictions of the model being built. Therefore, spectra data may be corrected using some pre-processing methods, including normalization, multiplicative scatter correction (MSC), standard normal variate (SNV), orthogonal signal correction (OSC), spectra derivatives, detrending, and combination of them. According to Pasquini [1], this selection of pre-processing shall follow with the knowledge of sample characteristics, sample measurement procedure, radiation interaction, and analytical issue requirements.

**Fig. 2:**
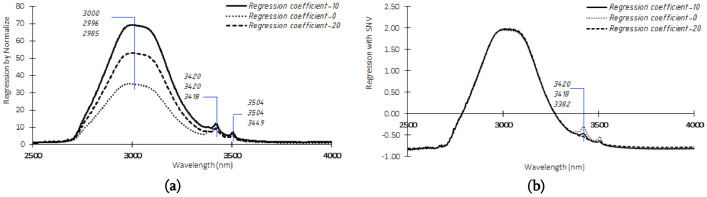
FTIR spectrum after pre-processing (a) coconut milk from traditional markets using pre-processing normalize and (b) instant coconut milk from modern market using pre-processing SNV.

Adulteration of coconut milk with water, can be seen by changing the values from the spectral data. Certain attributes reflecting the amount of light absorbed, reflected, or transmitted have corresponded with particular wavelengths. Spectra data can also be processed into its normalize for coconut milk from traditional markets and SNV for instant coconut milk from the modern market (Fig. 2) to identify spectra and find noises through the wavelength region deeply. Spectra derivatives are an excellent way to correct these distortions, commonly employing a Savitzky-Golay derivative algorithm [2]. After pre-processing a sample of coconut milk from the traditional market, it can be seen in Fig. 2a. It can be more clearly seen the difference in absorbance for each adulteration level for coconut milk. However, it is different from coconut milk from modern markets (Fig. 2b), which tends to have an overlapping absorption amount.

The purpose of using infrared spectral is to be able to predict and determine a chemical contained in the product. This approach is better known as the chemometrics method. In the past few years, there has been quite a lot of research related to the chemical content with its infrared spectral properties. Their focus is on how to be able to model this with great precision. Therefore, several linear multivariate regression approaches have been developed, which are often used to handle this, namely principal component regression (PCR) and partial least squares regression (PLSR), and some have even used artificial intelligence methods such as machine learning [3].

Two regression methods and seven spectra pre-processing were fitted to predict coconut milk adulteration (Tables 1 and 2). Coconut milk consists of two sources, viz comes from traditional markets and instant coconut milk. Both methods are trying to find the best correlation between FTIR spectra data and respective adulterations parameters. We can see that the PLSR regression methods with pre-processing normalize give the best correlation on calibration and validation for coconut milk from traditional markets. For instant coconut milk from the modern market, PLSR regression methods with SNV pre-processing provide the best correlation on calibration and validation correlation.

**Table 1:** Comparison of PCR and PLSR approach on coconut milk from traditional market.

			Calibration			Cross Validation	
Method	Spectra treatment	Number of LVs	r	R^2^	RMSE-C	r	R^2^
PCR	Original	*6*	*0.985*	*0.970*	*1.377*	*0.962*	*0.924*
	Normalize	*7*	*0.984*	*0.968*	*1.430*	*0.989*	*0.966*
	MSC	*6*	*0.962*	*0.925*	*2.181*	*0.978*	*0.921*
	SNV	*6*	*0.977*	*0.954*	*1.708*	*0.955*	*0.864*
	1st derivative	*7*	*0.922*	*0.849*	*3.100*	*0.759*	*0.583*
	2nd derivative	*2*	*0.574*	*0.329*	*6.542*	*0.774*	*0.461*
	1st derivative+MSC	*7*	*0.914*	*0.836*	*3.233*	*0.715*	*0.519*

PLSR	Origimal	*7*	*0.990*	*0.979*	*1.145*	*0.971*	*0.945*
	Normalize	*7*	*0.990*	*0.980*	*1.129*	*0.989*	*0.970*
	MSC	*6*	*0.989*	*0.977*	*1.207*	*0.988*	*0.906*
	SNV	*3*	*0.937*	*0.878*	*2.784*	*0.963*	*0.817*
	1st derivative	*5*	*0.997*	*0.994*	*0.613*	*0.859*	*0.716*
	2nd derivative	*2*	*0.605*	*0.366*	*6.361*	*0.751*	*0.462*
	1st derivative+MSC	*5*	*0.997*	*0.995*	0.572	*0.847*	*0.680*

**Table 2:** Comparison of PCR and PLSR approach on instant coconut milk from modern market.

			Calibration			Cross Validation	
Method	Spectra treatment	Number of LVs	r	R^2^	RMSE-C	r	R^2^
PCR	Original	*3*	*0.832*	*0.692*	*4.433*	*0.911*	*0.465*
	Normalize	*4*	*0.878*	*0.771*	*3.820*	*0.753*	*0.283*
	MSC	*6*	*0.960*	*0.922*	*2.227*	*0.940*	*0.842*
	SNV	*4*	*0.912*	*0.838*	*3.219*	*0.958*	*0.892*
	1st derivative	*6*	*0.827*	*0.685*	*4.484*	*0.196*	*0.016*
	2nd derivative	*1*	*0.149*	*0.022*	*7.897*	*0.570*	*0.159*
	1st derivative+MSC	*6*	*0.842*	*0.709*	*4.311*	*0.339*	*0.131*

PLSR	Origimal	*6*	*0.966*	*0.934*	*2.051*	*0.868*	*0.762*
	Normalize	*4*	*0.894*	*0.800*	*3.571*	*0.729*	*0.303*
	MSC	*5*	*0.965*	*0.931*	*2.097*	*0.967*	*0.923*
	SNV	*5*	*0.977*	*0.955*	*1.688*	*0.972*	*0.929*
	1st derivative	*5*	*0.984*	*0.968*	*1.422*	*0.488*	*0.280*
	2nd derivative	*1*	*0.232*	*0.054*	*7.768*	*0.432*	*0.155*
	1st derivative+MSC	*5*	*0.992*	*0.984*	1.022	*0.598*	*0.393*

**Fig. 3:**
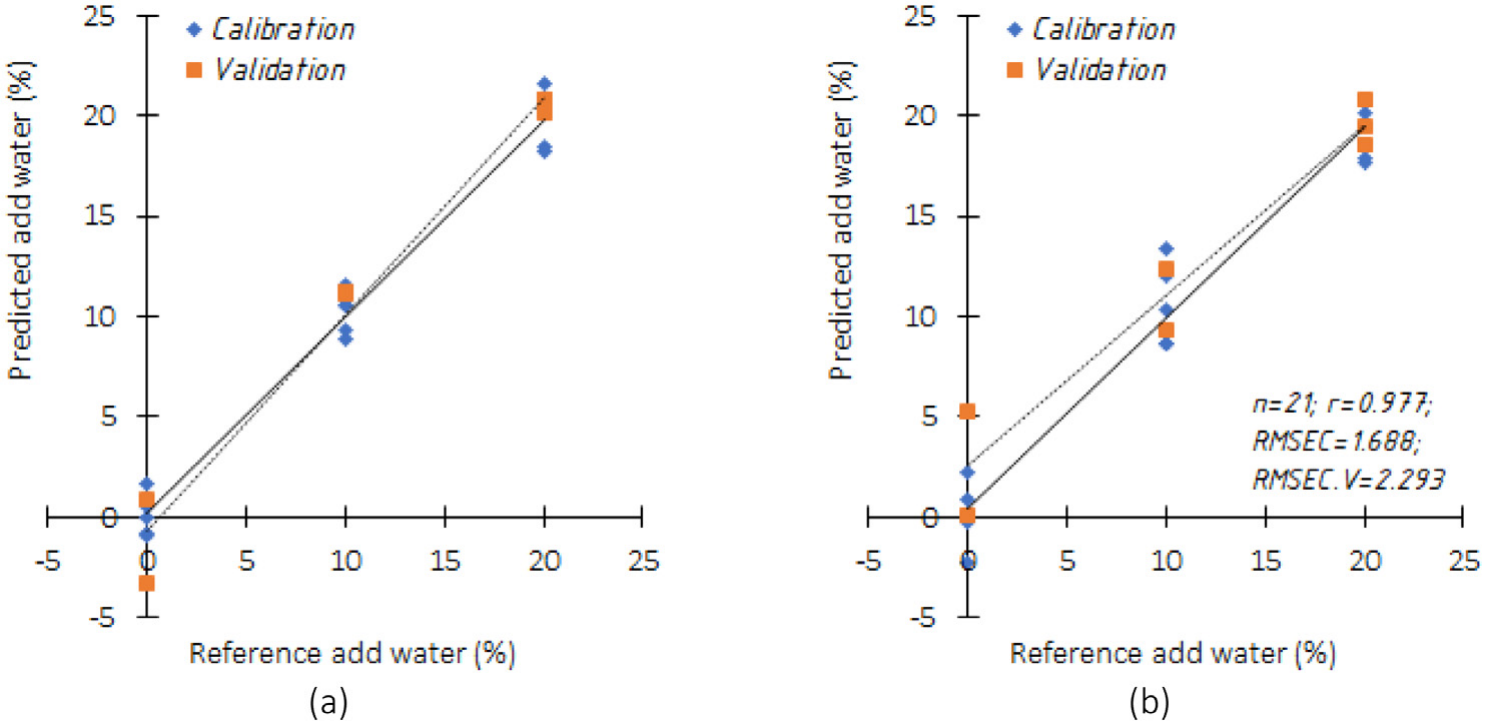
Scatter plot of reference vs. predicted by FTIR based on (a) normalize pre-processing and PLSR, and (b) SNV pre-processing and PLSR.

From the best correlation for coconut milk from traditional markets in Table 1 and instant coconut milk from modern markets in Table 2, a prediction model is developed. By regressing spectrum data and actual attributes obtained by standard laboratory procedures, prediction models were developed. As seen in Fig. 3, the study findings were then compared with actual results to determine the predicted accuracy. Based on the dataset, the model built has been able to predict adulteration for coconut milk from traditional markets with statistical indicators of (*r*) 99% (Fig. 3a). The predictive value of the model is preferable to the statistical indicators (*r*) for predicting adulteration of coconut milk from the modern market (Fig. 3b).

## 2 Experimental Design, Materials and Methods

### 2.1 Sample prepration

The samples tested were coconut milk consisting of coconut milk from traditional markets and instant coconut milk from modern markets. Each sample was prepared in conditions without adulteration (0%), 10% adulterated with water, and 20% adulterated with water with laboratory standards. A total of 7 samples were prepared from each adulteration treatment.

### 2.2 Instrument setup and spectra data acquisition

Infrared spectra data generated using a FTIR instrument (Bruker alpha II) in the wavenumber range from 3997 to 2500 cm^−1^ with 2.06 cm^−1^ resolution windows. The halogen lamp's light source irradiated coconut milk samples through a quartz window with 1 cm of diameter. Liquid sample coconut milk was placed manually in the crystal on top of the FTIR instrument. A total of 42 samples of coconut milk will be taken from spectra data. In order to identify coconut milk samples, sample labeling was required manually before spectra data collection for each spectra acquisition.

### 2.3 Infrared spectra data pre-processing

According to some research results, coconut milk infrared spectral raw data should be pre-processing before designing and developing models of calibration. This eliminates noise that includes insignificant background data and noise that can influence the adulteration of coconut milk and other consistency qualities and conflict with them. Pre-processing datasets of spectra were obtained by converting the data of spectra using specified algorithms: normalize, multiplicative scatter correction (MSC), standard normal variate (SNV), 1st derivative, 2nd derivative, and combination of 1st derivative and MSC. For spectral data from rice vinegar, pre-processing spectra with normalize works better [4]. Pre-processing multiplicative scatters correction (MSC), and standard normal variate (SNV) is also the most common correcting spectra and enhancement method used for intact grape berries [5]]. MSC is compensated for additive and multiplicative effects in the spectral data caused by physical effects [6]. The algorithm also tried to reduce scattering effects by linearising each spectrum to an optimal range of spectra data that fits the typical spectrum [7], [8], [9]. Besides that, SNV pre-processing works to normalize each spectrum of the spectra data to zero mean and unit variance. On the other hand, several researchers have tried to merge the two pre-processing approaches to establish improved calibration and prediction efficiency [10], [11], [12].

### 2.4 Prediction models

This paper's regression approaches are principal component regression (PCR) and partial least square regression (PLSR). The two methods, according to some research results, are powerful for regression in infrared spectroscopy applications. The two regression approaches will generate a latent variable (LVs) as a principal component and factor in PCR and PLSR.

Calibration models are formed with 2/3 of the acquired spectrum data. Cros validation uses 1/3 of the spectrum data another. The procedure for selecting data for calibration and validation in this manuscript is carried out randomly but still must represent the data from each test [13], [14], [15]. Although according to several research results related to the application and practice of managing spectral data, this is difficult to determine because it is related to potential data outliers. Rahmaddiansyah et al. [16] suggest using the principal component analysis (PCA) technique and applying Hotelling T2 ellipse to overcome this. Prediction performance is evaluated with the parameters correlation coefficient (r), coefficient of determination (R2), root mean square error in calibration (RMSEC). The higher both the value of correlation coefficient (r) and coefficient of determination (R2), the greater probability of models to predict adulteration from coconut milk dataset accurately (see Fig. 3).

## Declaration of Competing Interest

The authors declare that they have no known competing financial interests or personal relationships which have, or could be perceived to have, influenced the work reported in this article.

## Appendix Supplementary materials

Image, application 1
